# Unravelling Quality of Life for Head and Neck Cancer Patients after VMAT Radiation Therapy: Insights from Toxicity, Dosimetry and Symptoms Correlation

**DOI:** 10.3390/clinpract14030086

**Published:** 2024-06-06

**Authors:** Panagiota Kiafi, Maria Anthi Kouri, Georgios Patatoukas, Andromachi Kougioumtzopoulou, Marina Chalkia, Ourania Nicolatou-Galitis, Vassilis Kouloulias, Efthimios Kyrodimos, Kalliopi Platoni

**Affiliations:** 1 2nd Department of Radiology, Medical School, Attikon University Hospital, National and Kapodistrian University of Athens, 11527 Athens, Greece; mariakouri90@gmail.com (M.A.K.); gpatatouk@med.uoa.gr (G.P.); ankougio@med.uoa.gr (A.K.); chalkiamar@outlook.com (M.C.); vkouloul@med.uoa.gr (V.K.); 2 Oral Oncology Unit, Clinic of Hospital Dentistry, Dental School, University of Athens, Bouboulinas 41, N. Psyhico, 15451 Athens, Greece; onikolat@dent.uoa.gr; 3 2nd Department of Otolaryngology-Head and Neck Surgery, Hippokration General Hospital, University of Athens, 15451 Athens, Greece; ekirodim@med.uoa.gr

**Keywords:** quality of life, EORTC QLQ-C30, EORTC QLQ-H&N35, head and neck cancer, dosimetric toxicity, symptoms and toxicity, VMAT, radiation therapy, healthcare

## Abstract

(1) Background: Head and neck cancer treatment, including advanced techniques like Volumetric Modulated Arc Therapy (VMAT), presents challenges for maintaining patient quality of life (QoL). Thus, thoroughly investigating how radiation therapy (RT) affects patients has been proved essential. Derived by that, this study aims to understand the complex interactions between not only RT and QoL but also symptom severity, and treatment-related toxicities in three distinct time points of patient’s treatment; (2) Methods: To achieve that, EORTC-QLQ-C30 and EORTC QLQ-H&N35 questionnaires were used in combination with EORTC_RTOG scoring criteria and Spearman’s rho statistical analysis for 74 patients with cancer undergoing VMAT radiation therapy; (3) Results: The results revealed a significant improvement in the Overall Health Index post-treatment, indicating a temporary decline during therapy followed by subsequent recovery, often surpassing pre-treatment QoL levels. Concurrently a reduction in symptomatology was observed, notably in pain, swallowing difficulties, and dry mouth, aligning with prior research indicating decreased symptom burden post-treatment. However, Spearman’s correlation coefficient analysis at two distinct time points during therapy uncovered varying degrees of correlation between dosimetric data at Organs at Risk (OARs) and reported symptoms, highlighting potential limitations in using QoL questionnaires as sole indicators of treatment efficacy. Our investigation into the correlation between dosimetric data, toxicity, and symptoms focused on the relationship between radiation doses and oral mucositis levels, a common toxicity in head and neck cancer patients. Significant associations were identified between toxicity levels and dosimetric parameters, particularly with OARs such as the parotid glands, oral cavity, and swallowing muscles, underlining the utility of the EORTC method as a reliable toxicity assessment tool; (4) Conclusions: To summarize, current research attempts to underscore the importance of refining QoL assessments for enhanced patient care. The integration of dosimetric data, symptom severity, and treatment-related toxicities in the QoL outcomes of head and neck cancer patients undergoing VMAT radiation therapy, can lead towards the optimization of treatment strategies and the improvement of patient outcomes in future patient-centered radiation therapy practices.

## 1. Introduction

Head and neck cancer remains a significant health concern worldwide, with radiation therapy being a cornerstone in its treatment regimen and technique [1,2]. However, the impact of radiation therapy on patients’ quality of life (QoL) is a crucial aspect that necessitates thorough investigation [3,4].

The assessment of quality of life (QoL) in patients with cancer is paramount for understanding the holistic impact of treatment interventions. To this end, the European Organization for Research and Treatment of Cancer (EORTC) has developed two widely utilized instruments: the Quality of Life Questionnaire Core 30 (QLQ-C30) and the Head and Neck Cancer-Specific Module (QLQ-H&N35) [5]. These questionnaires offer a comprehensive evaluation of QoL domains, encompassing physical, role, emotional, cognitive, and social functioning, along with symptomatology such as fatigue, pain, and nausea/vomiting [6]. Specifically tailored to the head and neck cancer population, the QLQ-H&N35 module addresses symptoms pertinent to this patient cohort, including swallowing difficulties, speech problems, and body image concerns. These instruments provide invaluable insights into patients’ subjective experiences, aiding clinicians in tailoring treatment strategies to optimize QoL outcomes [7].

In conjunction with advancements in treatment modalities, such as volumetric-modulated arc therapy (VMAT) for head and neck cancer, which offers precise targeting of tumor volumes while sparing surrounding healthy tissues, the integration of QoL assessments becomes even more crucial. Compared to traditional radiotherapy techniques, VMAT allows for improved sparing of critical structures, thereby reducing treatment-related toxicities and further enhancing patients’ QoL during and after treatment [8]. This combination of advanced treatment methodologies and comprehensive QoL assessment tools represents a significant stride towards personalized cancer care, aiming not only for effective tumor control but also for the preservation of patients’ well-being and functional status.

Assessing the doses deposited in organs at risk (OARs) is paramount in modern radiotherapy planning, especially in the context of head and neck cancer treatment. The Quantitative Analyses of Normal Tissue Effects in the Clinic (QUANTEC) guidelines provide dose constraints for various anatomical structures, ensuring that radiation-induced toxicities are minimized while maintaining effective tumor control [9]. Even without exceeding these constraints, increased toxicity levels at OARs are potentially compromising patients’ QoL. Toxicities, such as oral mucositis and xerostomia, are common in head and neck cancer patients undergoing radiation therapy and can significantly impact their daily functioning and overall well-being [10,11]. To quantify and manage these toxicities, specific scoring systems such as the Radiation Therapy Oncology Group (RTOG) and European Organization for Research and Treatment of Cancer (EORTC) Late Radiation Morbidity Scoring Schema are utilized [12]. These scoring systems enable clinicians to objectively evaluate the severity of treatment-related toxicities, guide treatment modifications or supportive care interventions accordingly, and enhance patients’ overall treatment experience. Thus, the integration of dosimetric data, adherence to QUANTEC guidelines, and utilization of toxicity scoring systems play a pivotal role in optimizing treatment outcomes, minimizing adverse effects, and ultimately improving patients’ QoL during and after radiotherapy for head and neck cancer [13,14].

This paper is emphasizing into the integration of patient-reported outcomes, dosimetric data, and toxicity assessments in order to elucidate the intricate relationship between radiation therapy, QoL outcomes, symptoms severity and treatment-related toxicities in head and neck cancer patients. By delving into these multifaceted aspects, the aim is to shed light on pivotal questions surrounding the benefits of QoL questionnaires. The final endeavor is to provide insights into whether there exists a direct correlation between QoL metrics, symptomatology, and toxicity levels. Thus, by investigating such emerging inquiries pertinent to future clinical practice and their potential implications we are hoping into contributing to the refinement and optimization of patient care strategies in this challenging clinical domain.

## 2. Materials and Methods

### 2.1. Quality of Life Assessment

#### 2.1.1. Study Design

This study aimed to evaluate the quality of life (QoL) among 74 patients undergoing VMAT radiation therapy for head and neck cancer using the European Organization for Research and Treatment of Cancer Quality of Life Questionnaire Core 30 (EORTC QLQ-C30) and the Head and Neck Cancer-Specific Module (EORTC QLQ-H&N35). The study was conducted in accordance with the principles outlined in the Declaration of Helsinki and was approved by the Bioethics and Ethics Committee of the Scientific Council of the Attikon General University Hospital, Athens, Greece.

#### 2.1.2. Participants

Patients eligible for inclusion in this study were those diagnosed with head and neck cancer who were scheduled to undergo VMAT radiation therapy as part of their treatment regimen. Informed consent was obtained from all participants prior to enrollment in the study. The initial number of recruited patients reached that of 82. The exclusion criteria pertained change of prescribed therapy, patients requirement to be excluded from the study and diseased during treatment. 

#### 2.1.3. Data Collection and Analysis

Data collection was carried out at Attikon University Hospital, Athens, Greece, spanning from 18 January 2021 to 29 March 2022. Patient-reported outcomes regarding quality of life were evaluated using the EORTC QLQ-C30 and QLQ-H&N35 questionnaires. The EORTC QLQ-C30, a validated instrument, comprehensively assesses the quality-of-life domains in patients with cancer, including physical, role, emotional, cognitive, and social functioning, along with symptoms such as fatigue, pain, nausea/vomiting, dyspnea, insomnia, appetite loss, and gastrointestinal issues. Additionally, the EORTC QLQ-H&N35 module specifically addresses quality of life issues relevant to head and neck cancer patients, including symptoms like pain, swallowing difficulties, speech problems, social eating, and body image concerns. The questionnaires were administered at three distinct time points: (i) before initiating radiation therapy treatment, (ii) immediately after the completion of radiotherapy and (iii) three months after radiotherapy, providing a comprehensive understanding of the temporal evolution of quality-of-life outcomes throughout the treatment process. Moreover, by utilizing these two questionnaires, we were able to gather data on both the overall quality of life of patients and on the specific symptoms experienced by them before and after radiation therapy and thus, enriching our analysis and providing a more nuanced perspective on the impact of radiation treatment on patients’ well-being.

### 2.2. Dosimetric Data Collection and Assessment

#### 2.2.1. Dosimetric Data Acquisition

Dosimetric data for 74 patients was derived from the treatment planning system (Eclipse, Varian) (TPS)utilized for radiation therapy. The treatment planning procedure involved a comprehensive process aimed at optimizing radiation delivery to the tumor region, while minimizing adverse effects on the surrounding organs at risk (OARs). Each patient underwent computed tomography (CT) imaging for precise anatomical delineation, using an appropriate immobilization with 5-point thermoplastic head mask. Treatment plans were developed considering factors such as tumor location, size, and proximity to critical structures. Total doses in Gray (Gy) deposited in the OARs of the patients were meticulously assessed. 

The dosimetric parameters of interest for the anatomical regions of the OARs collected and analyzed for each patient pertained: (A) Average Dose (Gy) of left Parotid Gland, (B) Average Dose (Gy) of right Parotid Gland, (C) Average Dose (Gy) of Oral Cavity, (D) Average Dose (Gy) of Pharyngeal Constrictors, (E) Volume (cc) of Right Parotid Gland receiving dose > 26 Gy, (F)Percentage (%) of Right Parotid Gland Volume receiving dose > 26 Gy, (G) Volume (cc) of Left Parotid Gland receiving dose > 26 Gy, (H) Percentage (%) of Left Parotid Gland Volume receiving dose > 26 Gy, (I) Volume(cc) of Oral Cavity receiving dose > 30 Gy, (J) Percentage (%) of oral cavity Volume receiving dose > 30 Gy, (K) Total volume (cc) receiving dose > 107% of the prescribed dose, (L) Percentage (%) of volume receiving > 107% of the prescribed dose These parameters were determined according to the Quantitative Analyses of Normal Tissue Effects in the Clinic (QUANTEC) instructions, which provide dose constraints for various anatomical structures in head and neck cancer radiotherapy. These constraints aim to limit radiation-induced toxicities. Dosimetric data were analyzed to assess compliance with established dose constraints and to evaluate potential correlations between dosimetric parameters and patient-reported quality of life outcomes.

#### 2.2.2. Acquisition of Toxicity Data

The assessment of oral mucositis and dosimetric toxicity in this study was conducted through the integration of dosimetric data obtained from organs at risk (OARs) with established scoring systems developed by the European Organization for Research and Treatment of Cancer (EORTC) and the Radiation Therapy Oncology Group (RTOG). These dosimetric parameters were correlated with the presence and severity of oral mucositis as assessed by the EORTC_RTOG scoring criteria. By aligning dosimetric data with clinical outcomes such as mucositis severity, a comprehensive evaluation of the relationship between radiation dose deposition in OARs and treatment-related toxicities, particularly oral mucositis was allowed. The toxicity grading system employed in this study ranged from 0 to 4, with each grade reflecting increasing severity of treatment-related side effects, facilitating precise evaluation and management of patient outcomes. 

### 2.3. Statistical Analysis

#### 2.3.1. Quality of Life Data Correlation

Data obtained from the questionnaires were scored according to the guidelines provided by the EORTC QLQ-C30 and QLQ-H&N35 scoring manuals. The study identified quality of life percentages and progression for the three distinct time points as well as several key symptoms reported by head and neck cancer patients, including Physical, Global Health, Appetite Loss, Pain, Swallowing, Senses Problems, Dry Mouth, Sticky Saliva, and Weight Loss.

Statistical analysis was performed using IBM SPSS Statistics version 26.00 (IBM Corporation, Somers, NY, USA) to access the correlation of symptoms with the dosimetric data of radiation therapy. A significance level of *p* < 0.05 was considered statistically significant. Notably, the significance of symptoms was assessed separately, for the radiation effects immediately after the completion of treatment, and for the effects occurred three months after treatment by examining their correlation with the doses at the organs at risk (OARs). This approach allowed for a comprehensive evaluation of the relationship between symptomatology and radiation dose deposition, both immediately after treatment but in the 3 months post-treatment period too.

#### 2.3.2. Toxicity Data Correlation

Spearman’s rho statistical analysis was conducted to evaluate the correlation between toxicity grades and dosimetric values of the organs at risk (OARs), assessing both the observed correlation coefficient and the associated *p*-value. This statistical approach allowed for a comprehensive examination of the relationship between radiation dose deposition in OARs and the severity of treatment-related toxicities, providing insights into the potential impact of radiation therapy on patient outcomes. 

Similarly, Spearman’s rho statistical analysis was employed to assess the correlation coefficient and associated *p*-value between symptoms derived from Quality-of-Life questionnaires and toxicity grades. This analysis allowed for the exploration of the relationship between patient-reported symptoms and treatment-related toxicities, providing observations into the impact of radiation therapy on patient outcomes. By examining both correlations, the study gained a comprehensive understanding of the associations between dosimetric values, symptoms, and treatment-related toxicities, contributing to a more nuanced evaluation of the effects of radiation therapy on quality of life in head and neck cancer patients. 

The ranges and indications for the correlation coefficient (r), its size and interpretations are presented in Tables S1 and S2 both available in Supplementary Materials. Furthermore, English language in this paper has been edited and refined by AI.

## 3. Results

### 3.1. Quality of Life Data 

**Figure 1:**
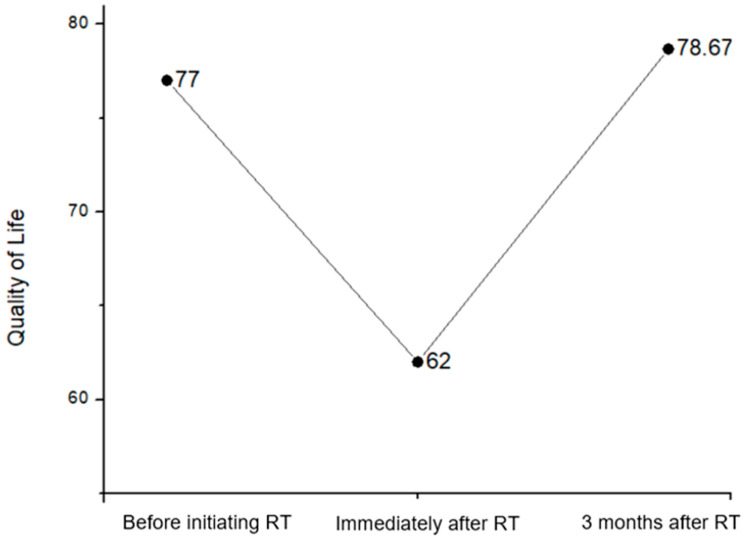
Quality of life of head and neck patients for the different time points before and after Radiation Therapy (RT) for the Overall health index of the EORTC QLQ-C30.

The QoL scores for the Overall health index of patients before the initiation of VMAT radiation therapy, immediately after, and 3 months following its completion was evaluated using the EORTC-QLQ-C-30 QoL scale were 77.00 (±17.27), 62.00 (±21.67) and 78.67 (±15.51), respectively as is depicted in Figure 1.

The statistical analysis of the over QoL score indicated a statistically significant difference between the longitudinal assessments of the Overall Health Index (*p* < 0.0005). Pairwise comparisons highlight a statistical difference among the 2nd and 1st measurements (*p* < 0.0005) and that of 2nd and the and 3rd (*p* < 0.0005) measurements respectively, while there is no significant difference between the 1st and 3rd measurements (*p* = 1.000).

**Figure 2:**
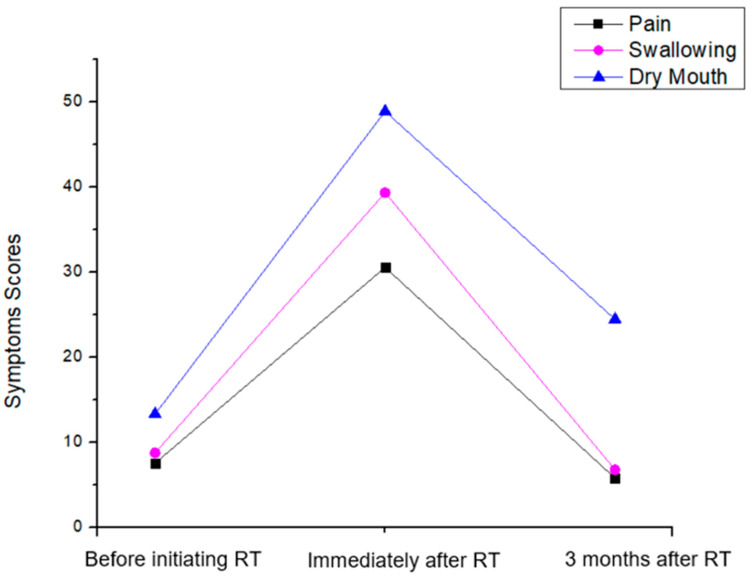
Symptom Scores of head and neck patients for the different time points before and after Radiation Therapy (RT) for the Pain, Swallowing Difficulties and Dry mouth indexes of the EORTC QLQ-C30.

The Symptoms scores of head and neck patients before radiation therapy, immediately after, and 3 months following its completion were also evaluated through the EORTC-QLQ-C-30 QoL scale and are depicted in Figure 2. The most prevalent of those symptoms are (i) Pain with scores 7.56 (±14.05), 30.56 (±24.82) and 5.78 (±12.40), (ii) Swallowing difficulties: 8.78 (±16.49), 39.33 (±30.00) and 6.78 (±13.61) and (iii) Dry mouth: 13.33 (±21.92), 48.89 (±32.11) and 24.44 (±24.71). It was observed that there is a statistically significant difference between the longitudinal assessments of all Symptom Indexes (*p* < 0.0005). The fact that there exists a statistically significant difference in the EORTC-QLQ 30 subscales scores is consistent with the existing literature, reinforcing the established understanding in the field [15].

The significance of symptoms collected from the QLQ-H&N35 questionnaires and their correlation with the dosimetric values of the organs at risk (OARs) are depicted in Table 1 and Table 2 for effects immediately post-treatment and for the effects three months post treatment respectively.

### 3.2. Dosimetric and Toxicity Data

**Figure 3:**
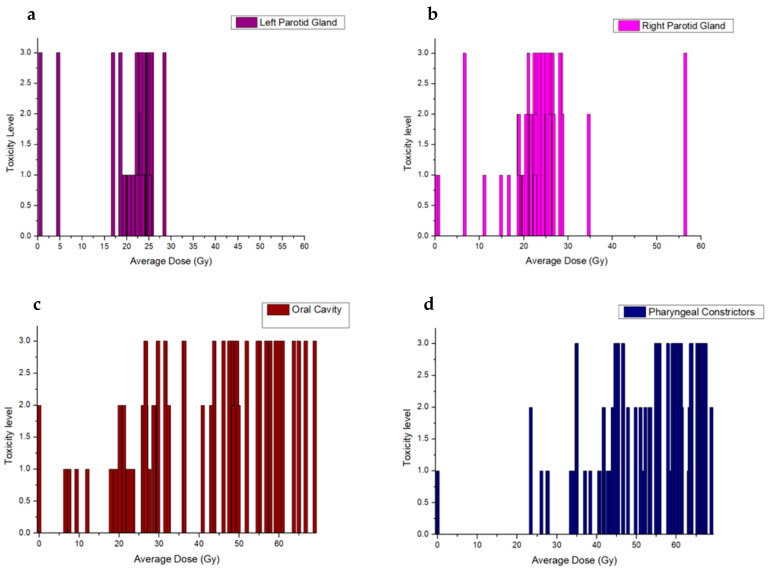
Toxicity levels (ranging from 0–4) for the deposited doses at (**a**) the OARs of Left Parotid Gland, (**b**) Right Parotid Gland, (**c**) Oral Cavity and (**d**) Pharyngeal Constrictors for head and neck cancer patiets after VMAT radiation therapy.

The assessment of toxicity due to oral mucositis and its levels correlated with the dosimetric parameters of the four major organs at risk (OARs) for the 74 head and neck patients with cancer is depicted in Figure 3.

### 3.3. Statistical Analysis

#### 3.3.1. Statistical Analysis of Dosimetric Data and Toxicity Correlation 

The Spearman’s rho statistical analysis for the correlation between toxicity grades and dosimetric values of the organs at risk (OARs) assessing both the observed correlation coefficient and the associated *p*-value depicted in Table 3. The high statistical correlation is marked with green. 

#### 3.3.2. Statistical Analysis of Toxicity and Symptoms Correlation

Similarly, the Spearman’s rho statistical analysis was conducted for the correlation among symptoms and dosimetric toxicity at OARs for the effects immediately after the completion of radiation treatment and the effects three months post treatment respectively (Table 4 and Table 5). 

## 4. Discussion

### 4.1. Quality of Life Assessment 

The evaluation of Quality of Life (QoL) and symptomatology in patients undergoing radiation therapy is promising to offer insights on the impact of treatment on their overall well-being. In this study, we utilized the EORTC-QLQ-C-30 QoL scale to assess changes in QoL scores and symptomatology among patients before, during, and after Volumetric Modulated Arc Therapy (VMAT) radiation therapy for head and neck cancers. It would be useful to mention that in our analysis of QoL related to tumor site, only patients with irradiated oropharyngeal or pharyngeal anatomical areas were included, as delineated by Patton et al. [16]. All patients underwent treatment using the same technique, VMAT-IGRT, ensuring consistency in the irradiation process. This uniformity is crucial for reducing variability in treatment related QoL outcomes. Furthermore, lymphopenia, which has been shown to impact QoL [17], was closely monitored. Baseline and ongoing laboratory evaluations included WBC assessments, and GCSF injections were promptly administered upon detection of lymphopenia to prevent prolonged episodes and associated complications such as mucositis. Additionally, the presence of oral candidiasis before irradiation, a factor known to exacerbate oral mucositis and negatively affect QoL [18], was addressed preemptively. All patients were evaluated by the Oral Oncology Department of the National and Kapodistrian University of Athens prior to the initiation of radiotherapy, ensuring that no cases of oral candidiasis were present at baseline. During radiotherapy, any occurrence of oral candidiasis was managed according to the MASCC/ISOO clinical practice guidelines for mucositis secondary to cancer therapy [19] primarily using oral antifungal treatments. This proactive management aimed to mitigate the impact on QoL and optimize patient outcomes throughout the treatment process.

Our results revealed a significant improvement in the Overall Health Index of patients over the course of treatment. Specifically, we observed a decrease in QoL scores immediately after the initiation of VMAT radiation therapy, followed by a subsequent increase three months post-treatment completion (Figure 2). This finding suggests that while patients may experience a temporary decline in QoL during treatment, they tend to regain and even surpass their pre-treatment QoL levels in the post-treatment period [4,20,21]. Furthermore, our analysis of symptomatology revealed a similar pattern of improvement over time. Patients reported a significant reduction in symptoms such as pain, swallowing difficulties, and dry mouth following completion of VMAT radiation therapy (Figure 3). These findings align with previous studies indicating that the Overall Health Index of patients is increasing over the course of treatment while the symptom burden tends to decrease post-treatment as acute side effects subside and patients adjust to long-term sequelae [4,20,21,22,23].

However, our analysis delved deeper into the significance of symptoms by conducting Spearman’s correlation coefficient analysis for the time points immediately after the completion of treatment and the one 3 months after it (Table 1 and Table 2 respectively). We found moderate statistical significance between certain symptoms (such as pain, swallowing difficulty, and dry mouth) and dosimetric data at the Organs at Risk (OARs) similarly to other research teams [24,25]. Conversely, symptoms like appetite loss, sticky mouth, and sensory problems showed low statistical correlation with dosimetric data. Interestingly, other symptoms exhibited no statistically significant correlation with doses at the OARs of the left and right parotid, oral cavity, and pharyngeal constrictor factors.

This raises concerns about the true benefits of QoL questionnaires in accurately correlating symptoms with radiation doses at OARs. The lack of consistent correlation suggests a potential limitation in using these questionnaires as sole indicators of treatment efficacy or toxicity. Further investigation into the reliability and validity of QoL questionnaires, alongside other objective measures, is warranted to enhance our understanding of treatment outcomes and guide clinical decision-making effectively.

### 4.2. Assessment of the Correlation of Dosimetric Data, Toxicity and Symptoms

#### 4.2.1. Correlation of Dosimetric Data and Toxicity

Following the correlation analysis of dosimetric data with symptoms, our investigation focused on the relationship between radiation doses and oral mucositis levels, a common toxicity in head and neck cancer patients. Utilizing dosimetric parameters of the four major Organs at Risk (OARs), we assessed toxicity levels for 74 patients as can be depicted in Figure 3. Our findings revealed a pattern in dose distribution among OARs, with lower doses observed for the parotids compared to the oral cavity. Of course this is highly dependent on the clinical case [26]. The parotids are rarely included in the Planning Target volume (PTV) and this is further explaining the low dose deposition. Contrary to that, structures like the oral cavity are included or surrounded by the PTV and thus it is increasing the difficulty of sparing the healthy tissue. Additionally, we identified a mild correlation between toxicity levels and radiation doses, indicating a tendency for increased toxicity with higher doses, albeit this relationship was highly dependent on the specific OAR involved, aligning with anticipated results [27]. 

The Spearman’s rho statistical analysis that we conducted to explore the correlation between toxicity grades and dosimetric values of Organs at Risk (OARs) provided the results presented in Table 1. This analysis allowed us to assess both the observed correlation coefficient and the associated *p*-value, providing insights into the relationship between treatment toxicity and radiation doses delivered to critical anatomical structures. Notably, our findings revealed moderate statistical correlations highlighted in blue, indicating significant associations between toxicity levels and dosimetric parameters. Specifically, we observed high positive correlations with OARs such as the left and right parotid glands, oral cavity, and swallowing muscles. Furthermore, indicators such as V > 26 Gy at the left parotid gland, V > 30 Gy at the oral cavity critical structure, and V > 30 Gy as a percentage of the oral cavity volume demonstrated significant correlations with treatment toxicity [28]. These dosimetric parameters were correlated with the presence and severity of oral mucositis as assessed by the EORTC_RTOG scoring criteria, highlighting the utility and sufficiency of the EORTC method as a reliable toxicity assessment tool. In general, the EORTC-RTOG criteria has been implemented in the clinical practice of radiation oncology for decades, by creating a “common language” among radiation oncologists throughout the world. They offer a standardized approach to grading treatment-related toxicities, ensuring consistency and comparability across studies, which allows for reliable data aggregation and meta-analyses [29]. These criteria cover a wide range of toxicity grades (0 to 4), capturing the full spectrum from no symptoms to severe conditions. This comprehensive grading is essential for a detailed understanding of treatment-related toxicities. The criteria reflect real-world clinical observations, making the toxicity data clinically relevant and applicable to everyday practice [30]. The detailed grading system facilitates precise evaluation and management of patient outcomes, allowing for tailored interventions and optimized treatment protocols. Furthermore, the EORTC-RTOG criteria have been validated in numerous studies, demonstrating their reliability and robustness in assessing radiation-induced toxicities [31]. The statistically significant correlation that was observed reinforces the validity of utilizing the EORTC_RTOG criteria in clinical practice for evaluating treatment-related toxicity, facilitating accurate monitoring and management of adverse effects in head and neck cancer patients undergoing radiation therapy. In summary, the EORTC-RTOG criteria provided a robust, standardized, and clinically relevant method for assessing treatment-related toxicities, allowing for comprehensive and precise evaluation and management of patient outcomes in our study. However, beyond the usefulness of the above scale, the introduction of QoL validated questionnaires might also be a missing chain in monitoring and follow-up of patients with cancer.

#### 4.2.2. Correlation of Toxicity and Symptoms

In our statistical assessment of the correlation between toxicity and symptoms at two distinct time points post-treatment, we observed varying degrees of correlation, highlighting potential limitations in utilizing Quality of Life (QoL) questionnaires as indicators of such correlations [32]. Specifically, for the first time point immediately after treatment, we identified only moderate positive correlation with the symptom of pain, as shown in Table 4. Conversely, at the second time frame, three months post-treatment, we found low positive correlations with indicators such as appetite loss, pain, and dry mouth. However, these correlations were not statistically significant, indicating a disconnect between treatment-related toxicity and reported symptoms through QoL questionnaires. This raises critical questions regarding the reliability and relevance of incorporating QoL assessments into clinical practice for oncology and radiation therapy of patients with cancer [33]. Should these results be considered in treatment decision-making, or is further evaluation and refinement necessary to ascertain their clinical significance? These findings underscore the need for continued research and scrutiny to elucidate the true value and utility of QoL questionnaires in guiding patient care effectively.

#### 4.2.3. Limitations and Future Perspectives

The findings of our study may be subjected to limitations associated with the nature of observational research. Firstly, a limitation regarding the sample size needs to be acknowledged. The sample size during participant recruitment indeed constitutes a factor that could affect the generalizability of the results and thus is a major concern in the majority of studies focusing on clinical regimes. While similar numbers of patients have been previously employed in analogous studies [24,34], the possibility of enlarging the sample size in future research endeavors is under consideration. Additionally, the fact that QoL questionnaires are relying on self-reported data introduces the possibility of bias in symptoms severity on behalf of the patient. Moreover, it is important to take into consideration that confounding variables such as the socioeconomic status, education level, age or psychological status of a cancer patient undergoing therapy may influence the relationships between QoL and symptoms severity. Lastly, the treatment heterogeneity within the study population, including treatment planning in VMAT radiation therapy, anatomic region of the malignancy as well as the progression of the disease in each individual may impact the interpretation of our findings. These limitations emphasize the need for cautious interpretation and suggest areas for future research to address potential biases. Furthermore, considering the heterogeneity in the nature of cancer, further research in various cancer types apart from head and neck could be beneficial towards a more conclusive correlation of QoL, dosimetric toxicity and symptoms after radiation therapy. 

Looking ahead, our research suggests that future advancements in patient-centered radiation therapy practices should focus on combining several evaluation indicators. This includes the comprehensive integration of QoL assessments, dosimetric data, and treatment-related toxicities. According to our results, we have noted interesting associations between dosimetric values and QoL parameters. Thus the insertion of QoL evaluation though validated questionnaires might be important for monitoring the impact of radiation induced toxicity in head and neck patients. By refining these assessments, we can better tailor treatment plans to individual patient needs, ultimately improving QoL outcomes and reducing the symptom burden for head and neck cancer patients undergoing radiation therapy. This multifaceted approach will pave the way for more precise and personalized treatment strategies, ensuring optimal care and better long-term outcomes for patients.

## 5. Conclusions

By employing the EORTC-QLQ-C30 QoL scale and the Head and Neck Cancer-Specific Module (EORTC QLQ-H&N35), we meticulously assessed changes in QoL scores and symptomatology before, during, and after Volumetric Modulated Arc Therapy (VMAT) radiation therapy. Notably, our findings unveiled a significant improvement in the Overall Health Index of patients’ post-treatment, indicating a transient decline during therapy followed by subsequent recovery or even surpassing pre-treatment QoL levels. Concurrently, we observed a parallel decrease in symptomatology, particularly in pain, swallowing difficulties, and dry mouth, aligning with previous studies indicating a reduction in symptom burden post-treatment. In addition to assessing the overall impact of radiation therapy on Quality of Life (QoL) outcomes and symptomatology, our analysis focused on the significance of symptoms by conducting Spearman’s correlation coefficient analysis for two distinct time points: immediately after treatment completion and three months thereafter. This detailed examination revealed varying degrees of correlation between dosimetric data at OARS and reported symptoms, highlighting potential limitations in utilizing QoL questionnaires alone as indicators of treatment efficacy.

In conjunction with our analysis of the correlation between dosimetric data, toxicity, and symptoms, we further investigated the relationship between radiation doses and oral mucositis levels, a common toxicity in head and neck cancer patients. By utilizing dosimetric parameters of the four major Organs at Risk (OARs), we assessed toxicity levels for 74 patients, revealing a discernible pattern in dose distribution among OARs. Specifically, we observed lower doses for the parotids compared to the oral cavity, which aligns with anticipated results. Moreover, our Spearman’s rho statistical analysis provided insights into the correlation between toxicity grades and dosimetric values of OARs, highlighting significant associations between toxicity levels and dosimetric parameters. Notably, we identified high positive correlations with OARs such as the left and right parotid glands, oral cavity, and swallowing muscles, indicating the utility and sufficiency of the EORTC method as a reliable toxicity assessment tool. These findings underscore the importance of integrating dosimetric data and toxicity assessments to optimize treatment outcomes and facilitate accurate monitoring and management of adverse effects in head and neck cancer patients undergoing radiation therapy.

To sum up, the intricate dynamics between radiation therapy, Quality of Life (QoL) outcomes and symptom severity were further verified. By adding the parameters of treatment-related toxicities in head and neck cancer patients and by integrating dosimetric data, the significance of refining QoL assessments for enhanced patient care is intensively highlighted. Looking ahead, this research findings are intending to add to the path of future advancements in patient-centered radiation therapy practices.

## Figures and Tables

**Table 1:** Statistical significance of symptoms collected from the QLQ-H&N35 questionnaires and their correlation with the dosimetric values of the organs at risk (OARs) immediately post-treatment.

0 Time Post RT		Physical	Global Health	Appetite Loss	Pain	Swallowing	Senses Problems	Dry Mouth	Sticky Saliva	Weight Loss
Left parotid gland	SCC	−0.221	−0.232	0.232	0.450	0.237	0.090	0.400	0.290	0.214
	*p*-value	0.065	0.065	0.065	<0.0005	0.055	0.459	0.001	0.015	0.075
Right parotid gland	SCC	−0.135	−0.141	0.160	0.347	0.104	0.053	0.244	0.107	0.129
	*p*-value	0.269	0.249	0.188	0.003	0.396	0.664	0.043	0.383	0.290
Oral cavity	SCC	−0.200	−0.149	0.252	0.432	0.307	0.203	0.415	0.197	−0.120
	*p*-value	0.126	0.254	0.052	0.001	0.017	0.120	0.001	0.131	0.361
Pharyngeal constrictor	SCC	−0.141	−0.151	0.240	0.323	0.236	0.256	0.404	0.175	0.095
	*p*-value	0.245	0.212	0.045	0.006	0.049	0.032	0.001	0.148	0.434

**Table 2:** Statistical significance of symptoms collected from the QLQ-H&N35 questionnaires and their correlation with the dosimetric values of the organs at risk (OARs) three months post treatment.

3 Months Post RT		Physical	Global Health	Appetite Loss	Pain	Swallowing	Senses Problems	Dry Mouth	Sticky Saliva	Weight Loss
Left parotid gland	SCC	0.008	−0.076	0.209	0.169	0.047	0.085	0.168	0.051	0.154
	*p*-value	0.948	0.534	0.083	0.161	0.699	0.482	0.164	0.675	0.202
Right parotid gland	SCC	−0.136	−0.181	0.163	0.341	0.180	−0.054	0.131	0.049	0.376
	*p*-value	0.264	0.137	0.182	0.004	0.140	0.657	0.284	0.689	0.001
Oral cavity	SCC	−0.237	−0.175	0.455	0.276	0.343	0.203	0.393	0.140	0.250
	*p*-value	0.068	0.180	<0.0005	0.033	0.007	0.121	0.002	0.287	0.054
Pharyngeal constrictor	SCC	−0.133	−0.166	0.318	0.223	0.198	0.265	0.339	0.210	0.154
	*p*-value	0.272	0.170	0.007	0.063	0.101	0.026	0.004	0.082	0.202

**Table 3:** Spearman’s rho statistical analysis for the correlation between toxicity grades and dosimetric data of the organs at risk (OARs).

Correlation of Toxicity Levels with Dosimetric Data at OARs		
SCC *		*p*-Value
Left Parotid Gland	0.641	<0.0005
Right Parotid Gland	0.594	<0.0005
Oral Cavity	0.839	<0.0005
Pharyngeal Constrictors	0.606	<0.0005
V > 26 Gy Right Parotid Gland CC	0.073	0.555
V > 26 Gy Right Parotid Gland %	0.198	0.109
V > 26 Gy Left Parotid Gland CC	0.215	0.078
V > 26 Gy Left Parotid Gland %	0.421	<0.0005
V > 30 Gy Oral Cavity CC	0.776	<0.0005
V > 30 Gy Oral Cavity %	0.724	<0.0005

* SCC: Spearman’s correlation coefficient.

**Table 4:** Spearman’s rho statistical analysis for the correlation among symptomatology and dosimetric toxicity at OARs for the effects immediately after the completion of radiation treatment.

Correlation of Toxicity Levels with Symptoms Immediately after Radiation Treatment		
SCC		*p*-Value
Physical	−0.195	0.094
Global Health	−0.176	0.130
Appetite Loss	0.160	0.170
Pain	0.454	<0.0005
Swallowing	0.281	0.015
Senses Problems	0.042	0.721
Dry Mouth	0.264	0.022
Sticky Saliva	0.174	0.136
Weight Loss	0.004	0.970

**Table 5:** Spearman’s rho statistical analysis for the correlation among symptomatology and dosimetric toxicity at OARs for the effects three months post radiation treatment.

Correlation of Toxicity Levels with Symptoms 3 Months after Radiation Treatment		
SCC		*p*-Value
Physical	−0.040	0.730
Global Health	−0.137	0.240
Appetite Loss	0.298	0.009
Pain	0.220	0.058
Swallowing	0.183	0.117
Senses Problems	0.090	0.444
Dry Mouth	0.240	0.038
Sticky Saliva	0.129	0.271
Weight Loss	0.111	0.344

## Data Availability

Data are available from the corresponding author within reasonable request.

## Supplementary Materials

The following supporting information can be downloaded at: https://www.mdpi.com/article/10.3390/clinpract14030086/s1, Table S1: The level of size of the correlation and its interpretation; Table S2: The level of correlation Coefficient (r) and its interpretation.
