# Supplementary material for: Unravelling Quality of Life for Head and Neck Cancer Patients after VMAT Radiation Therapy: Insights from Toxicity, Dosimetry and Symptoms Correlation
Source: Clin Pract. 2024 Jun 6;14(3):1085–99. doi: 10.3390/clinpract14030086 (PMC11202948; doi:10.3390/clinpract14030086)
Supplement: Supplementary file 1 [file clinpract-14-00086-s001.zip › clinpract-2989299-supplementary.pdf]

Supplementary Material

Unravelling Quality of Life for Head and Neck Cancer Patients after VMAT Radiation Therapy: Insights from Toxicity, Dosimetry and Symptoms Correlation

Panagiota Kiafi [1,*,†], Maria Anthi Kouri [1,†], Georgios Patatoukas [1], Andromachi Kougioumtzopoulou [1], Marina Chalkia [1], Ourania Nicolatou-Galitis [2], Vassilis Kouloulias [1], Efthimios Kyrodimos [3] and Kalliopi Platoni [1,*]

Table S1 The level of size of the correlation and its interpretation.

Size of Correlation	Interpretation
0.90 to 1.00 (−0.90 to −1.00)	Very high positive (negative) correlation
0.70 to 0.90 (−0.70 to −0.90)	High positive (negative) correlation
0.50 to 0.70 (−0.50 to −0.70)	Moderate positive (negative) correlation
0.30 to 0.50 (−0.30 to −0.50)	Low positive (negative) correlation
0.00 to 0.30 (0.00 to −0.30)	negligible correlation

Table S2 The level of correlation Coefficient (r) and its interpretation

Correlation Coefficient (r)	Interpretation
+1.0	Perfect positive
+0.8 to 1.0	Very strong
+0.6 to 0.8	Strong
+0.4 to 0.6	Moderate
+0.2 to 0.4	Weak
0.0 to +0.2	Very weak or no association
